# Glutamatergic stimulation induces GluN2B translation by the nitric oxide-Heme-Regulated eIF2α kinase in cortical neurons

**DOI:** 10.18632/oncotarget.11417

**Published:** 2016-08-19

**Authors:** Eva Ramos-Fernández, Marta Tajes, Gerard ILL-Raga, Lina Vargas, Arnau Busquets-García, Mònica Bosch-Morató, Biuse Guivernau, Victòria Valls-Comamala, Maria Gomis, Cristina Grau, César Fandos, Mark D. Rosen, Michael H. Rabinowitz, Nibaldo Inestrosa, Rafael Maldonado, Xavier Altafaj, Andrés Ozaita, Alejandra Alvarez, Rubén Vicente, Miguel A. Valverde, Francisco J. Muñoz

**Affiliations:** ^1^ Laboratory of Molecular Physiology, Faculty of Health and Life Sciences, Universitat Pompeu Fabra, Barcelona, Catalonia, Spain; ^2^ Cell Signaling Laboratory, Department of Cellular and Molecular Biology, Faculty of Biological Science, Pontificia Universidad Católica, Santiago, Chile; ^3^ Neuropharmacology Laboratory, Faculty of Health and Life Sciences, Universitat Pompeu Fabra, Barcelona, Catalonia, Spain; ^4^ Janssen Research and Development, L.L.C., San Diego, CA, United States of America; ^5^ CARE, Department of Cellular and Molecular Biology, Faculty of Biological Science, Pontificia Universidad Católica, Santiago, Chile; ^6^ Bellvitge Biomedical Research Institute, Unit of Neuropharmacology and Pain, University of Barcelona, Barcelona, Spain

**Keywords:** GluN2B, HRI kinase, nitric oxide, synapse, translation

## Abstract

The activation of N-Methyl D-Aspartate Receptor (NMDAR) by glutamate is crucial in the nervous system function, particularly in memory and learning. NMDAR is composed by two GluN1 and two GluN2 subunits. GluN2B has been reported to participate in the prevalent NMDAR subtype at synapses, the GluN1/2A/2B. Here we studied the regulation of GluN2B expression in cortical neurons finding that glutamate up-regulates GluN2B translation through the action of nitric oxide (NO), which induces the phosphorylation of the eukaryotic translation initiation factor 2 α (eIF2α). It is a process mediated by the NO-heme-regulated eIF2α kinase (HRI), as the effect was avoided when a specific HRI inhibitor or a HRI small interfering RNA (siHRI) were used. We found that the expressed GluN2B co-localizes with PSD-95 at the postsynaptic ending, which strengthen the physiological relevance of the proposed mechanism. Moreover the receptors bearing GluN2B subunits upon NO stimulation are functional as high Ca^2+^ entry was measured and increases the co-localization between GluN2B and GluN1 subunits. In addition, the injection of the specific HRI inhibitor in mice produces a decrease in memory retrieval as tested by the Novel Object Recognition performance. Summarizing our data suggests that glutamatergic stimulation induces HRI activation by NO to trigger GluN2B expression and this process would be relevant to maintain postsynaptic activity in cortical neurons.

## INTRODUCTION

Glutamatergic activation of N-Methyl D-Aspartate Receptor (NMDAR) induces intracellular signalling pathways with short-term effects such as local protein translation, and long-term effects that include changes in gene expression [1]. These effects are the basis for long term potentiation and synaptic plasticity, the processes underlying memory and learning in the cortex and hippocampus [2, 3].

The functional NMDAR is a heterotetrameric complex mainly composed of two GluN1 subunits together with either two GluN2 subunits or a combination of GluN2 and GluN3 subunits [4]. The GluN2B is the most pleiotropic subunit since it is crucial during brain development and also for mature neuronal function [5, 6]. In fact, recent evidence indicates that triheterotrimeric GluN1/2A/2B receptors could be the predominant NMDAR subtype at synapses of the adult hippocampus [7, 8], and may contribute to synaptic responses in other cortical and subcortical regions [9]. Functionally the GluN2B subunit interacts directly with the postsynaptic density-95 (PSD-95) scaffold proteins and, through PSD-95, with other key proteins involved in glutamatergic transmission such as neuronal nitric oxide (NO) synthase (nNOS) [10].

The regulation of the GluN2B subunit expression is performed at the translational level since its mRNA sequence has a long 5′untranslated region (UTR) containing three upstream AUG (uAUG), which is tigthly controlled by the eukaryotic translation initiation factor 2α (eIF2α). Under normal conditions mRNAs with long 5′UTR, GC-rich content, extensive secondary structure, upstream uAUGs and upstream open reading frames (ORF) show low efficiency of translation [11, 12]. Under special conditions such as stress or dendritic synapse activation, eIF2α is phosphorylated at serine (Ser) 51 by different kinases, resulting in reduced global translation [13, 14]. Paradoxically, transcripts containing multiple uORFs in their 5′UTRs, such as GluN2B, are translationally activated by phosporylation of eIF2α due to the relief of uORF-mediated inhibition [15-18].

Heme-regulated eIF2α kinase (HRI) is an enzyme recently reported to phosphorylate eIF2α in [18, 19]. HRI is activated following the interaction of NO with its heme group, inducing a transient phosphorylation of eIF2α. Once it is phosphorylated, it activates the translation of mRNAs containing specific 5′UTRs, which produce proteins with effects in the consolidation of object recognition memory [18,19]. Here we provide evidence for GluN2B translational de-repression following glutamatergic stimulation and activation of the NO-HRI-eIF2α phosphorylation signalling cascade in the cortex, providing the rapid protein translation required for neuronal activity, spine growth and memory formation.

## RESULTS

### Glutamatergic stimulation upregulates GluN2B expression by NO action

**Figure 1:**
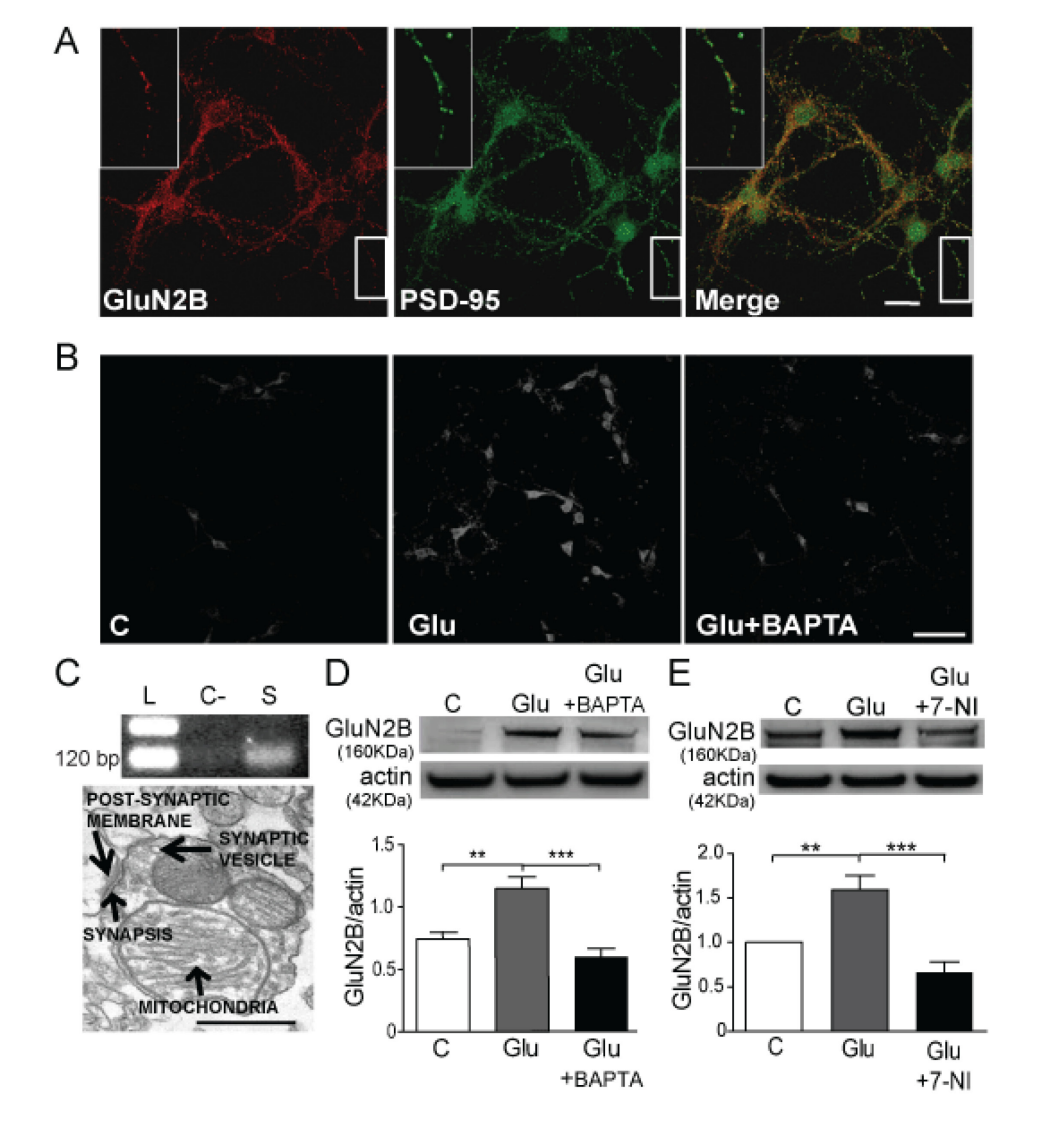
Glutamate increases GluN2B expression through a mechanism that involves calcium and NO **A.** Confocal immunofluorescence images of GluN2B (red) and PSD-95 (green) colocalization (merge) (Scale bar: 10 μm). **B.** GluN2B immunodetection in cultured mouse cortical neurons untreated (C: control) treated with 10 μM glutamate (Glu) for 1 h in the absence/presence of 10 μM BAPTA-AM (Glu+BAPTA) (Scale bar: 60μm). **C.** Agarose gel showing the cDNA of GluN2B subunit extracted from mouse cortical synaptosomes (L: ladder, C-: negative control without cDNA, S: cDNA from synaptosomes) and electron microscopy image of synaptosomes showing the active synaptic areas (Scale bar: 200nm). **D.** and **E.** WB from cortical mouse synaptosomes treated with 10 μM glutamate for 1 h in the absence and the presence of 10 μM BAPTA-AM, or 10 μM 7-NI and the respective quantification of GluN2B bands after correction by actin. Data are the mean ± SEM of 5 independent experiments. **p < 0.001 and **p < 0.0001 by one way ANOVA with Bonferroni's post-test.

We studied the GluN2B expression in primary neuronal cultures at DIV10 since it is the developmental stage when synapses on dendritic spines are detected [20]. We observed that GluN2B co-localizes with PSD-95 at the synaptic regions in mouse cortical neurons (Figure 1A). Similar results were reported in rat cortical neurons at DIV10-11 [21]. The Mander's coefficient (Supplementary Figure 1) shows that PSD-95 to GluN2B signal co-localizes at 30% and GluN2B to PSD-95 at 25%, indicating that GluN2B has a relevant synaptic expression. The synaptic location of GluN2B has been also demonstrated in synaptosomes from mouse cortex where GluN2B protein (Figures 1D, 1E) and mRNA (Figure 1C) are present.

Cortical neurons treated with glutamate for 1 h show an increase of GluN2B expression (Figure 1B) and this increase was prevented by the co-incubation with the Ca^2+^ chelator BAPTA·AM (Figure 1B). The same results were obtained in cortical synaptosomes (Figure 1D), a most controlled synaptic system. These results suggest that glutamate-induced GluN2B expression is dependent on Ca^2+^ signalling.

**Figure 2:**
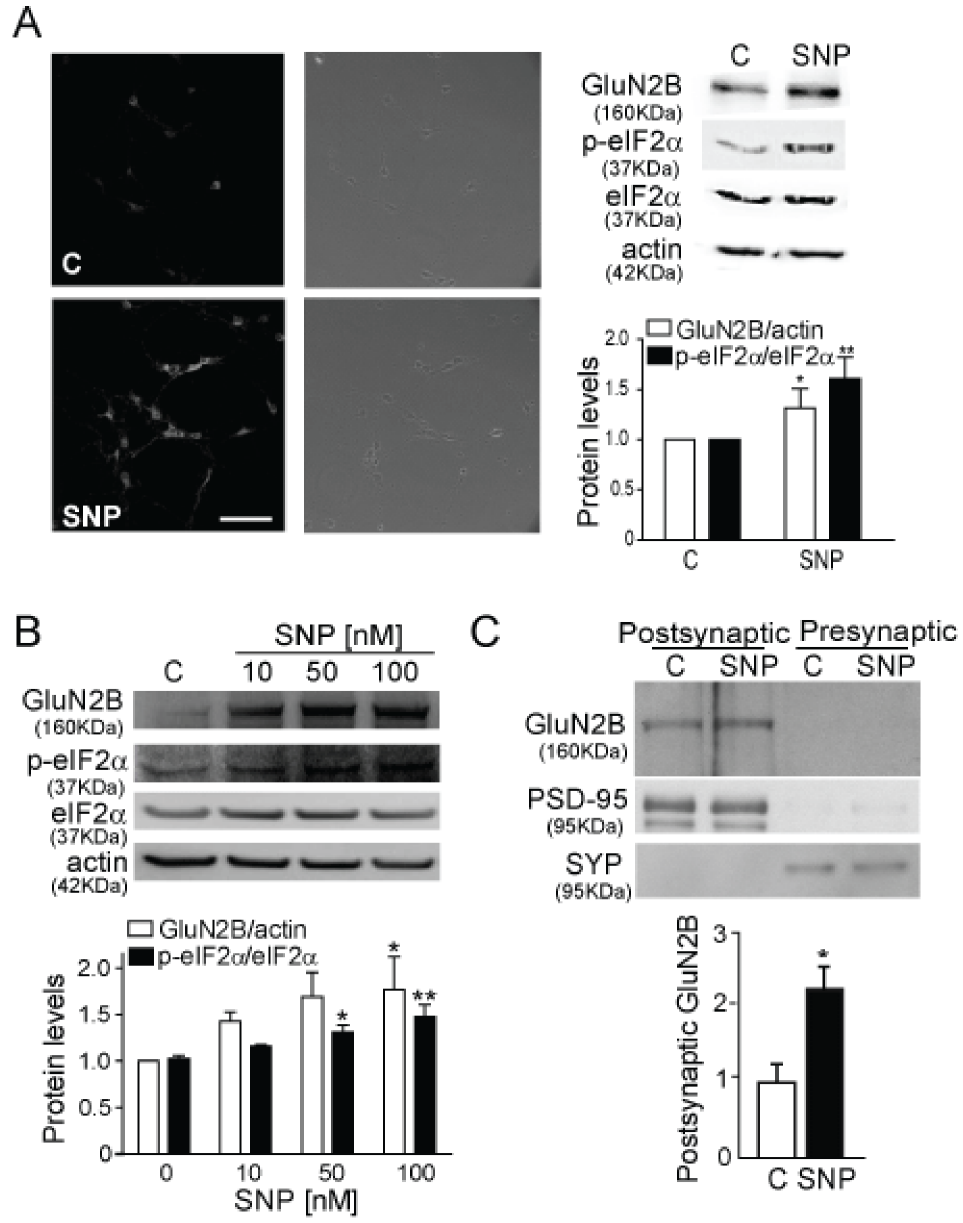
NO induces GluN2B expression and eIF2α phosphorylation **A.** GluN2B detection in cultured mouse cortical neurons treated with 100 nM SNP for 1 h by IF and WB (Scale bar: 60μm). The quantification of GluN2B and p-eIF2α are corrected respectively by actin and total eIF2α. Data are the mean ± SEM of 19 independent experiments. *p < 0.05, **p < 0.001 by Student *t*- test. **B.** WB from cortical mouse synaptosomes treated with 10, 50 and 100 nM SNP and band quantification after correction by actin and total eIF2α. Data are the mean ± SEM of 4 independent experiments. *p < 0.05 and **p < 0.001 by one-way ANOVA with Bonferroni's post-test. **C.** GluN2B detection in postsynaptic and presynaptic membrane from mouse cortical neurons treated with 100 nM SNP for 1 h. Synaptophysin/p38 (SYP) and PSD-95 are used to confirm the accuracy of the subcellular fractionation procedure. The histogram corresponds to the average of relative GluN2B expression levels in the PSD fraction. Data are the mean ± SEM of 3 independent experiments. *p < 0.05 by Student *t*- test.

NO is a very important agent in glutamatergic signalling and its production is dependent on Ca^2+^ [22], since it activates nNOS. We hypothesized that the downstream signalling cascade implicated in GluN2B increased expression may involve the production of NO, therefore we treated cortical synaptosomes with glutamate plus 7-nitroindazole (7-NI), a nNOS inhibitor (Figure 1E). We found that nNOS inhibition significantly prevented the glutamate-induced increase in GluN2B expression (*p* < 0.0001). In order to elucidate the role of NO we used SNP, a NO donor, to treat cortical neurons (Figure 2A) and we consistently found a significant increase in GluN2B expression (*p* < 0.05). The same results were obtained in cortical synaptosomes in a concentration-dependent manner being significant at 100 nM SNP (Figure 2B). The expression of GluN2B was also studied in isolated postsynaptic membranes from mouse cortical neurons (Figure 2C), confirming that NO increases GluN2B expression (*p* < 0.05) and this subunit can reach the postsynaptic membrane.

### NO controls GluN2B translation

Our findings suggest a rapid translation of the GluN2B subunit as a mechanism to control its synaptic expression. The eIF2α is a factor involved in the initiation of the translation and its phosphorylation is associated with the global arrest of translation [13, 14]. Paradoxically the phosphorylated eIF2α (p-eIF2α) levels were found to be increased by the exposure to SNP (Figures 2A, 2B).

**Figure 3:**
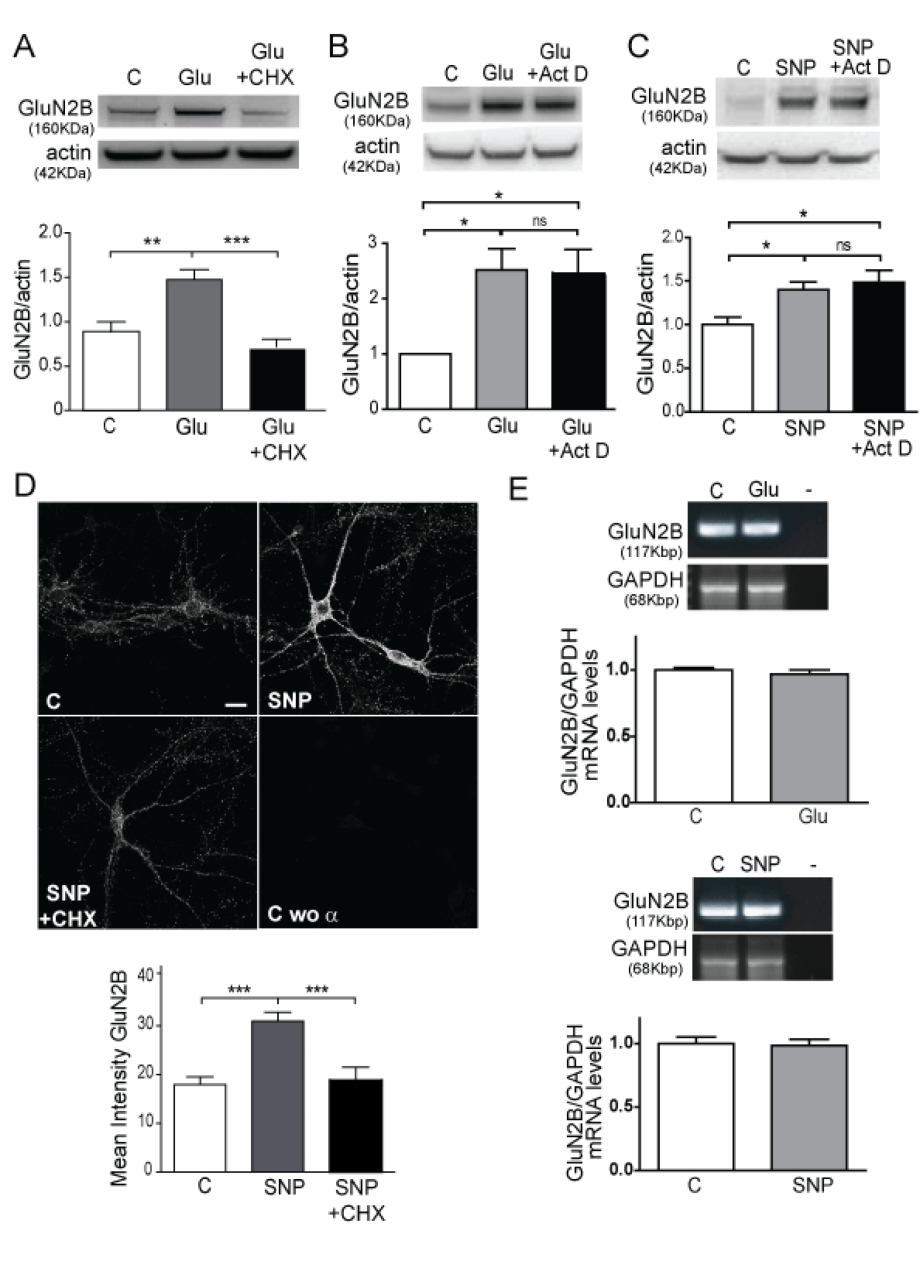
Glutamate and NO induce GluN2B translation **A.** WB of synaptosomes treated with 10 μM glutamate or 10 μM glutamate plus 100 μM CHX for 1 h. Quantification of GluN2B was corrected by actin. Data are the mean ± SEM of 5 independent experiments. **p < 0.001, ***p < 0.0001 by one-way ANOVA with Bonferroni's post-test. **B.** and **C.** WB of primary cortical cells treated with 10 μM glutamate or 100 nM SNP in the presence/absence of 1 μM Actinomicin D. Quantification of GluN2B was corrected by actin. Data are the mean ± SEM of 3 independent experiments. *p < 0.05 by one-way ANOVA with Newman-Keuls Multiple Comparison post-test. **D**. GluN2B detection by IF in cortical mouse neurons treated with 100 nM SNP in the presence/absence of 100 μM CHX for 1 h (Scale bar: 10μm). Mean Intensity of GluN2B are the mean ± SEM of 3 independent experiments (lower panel). ***p < 0.0001 by one-way ANOVA with Bonferroni's post-test. **E.** PCR images showing *GluN2B* and *GAPDH* expression of cells treated with 10 μm glutamate or 100 nM SNP. Quantification of *GluN2B* was corrected by *GAPDH*. Data are the mean ± SEM of 3 independent experiments statistically analyzed by Student *t*- test.

Cortical synaptosomes were treated with glutamate in the presence of CHX, a specific inhibitor of translation (Figure 3A). A significant impairment of the glutamate effect on GluN2B expression was obtained (*p* < 0.0001). Moreover when cortical neurons were treated with SNP in the presence of CHX (Figure 3D) the same impairment measured by mean intensity of GluN2B was obtained by immunofluroescence (*p* < 0.0001). These data strongly suggest that the induction of GluN2B expression by the glutamate-NO pathway is dependent on the translation of the protein. We discard the effect of transcription in the GluN2B increase because we perform the treatments for brief periods of time (1 h) and the study of GluN2B expression in synaptosomes, isolated synaptic vesicles, that have no nuclei, eliminates the transcription as an active mechanism in the studied signalling. In order to completely discard transcription as a possible mechanism affecting our results, we treated the cells with the transcription inhibitor Actinomycin D (Act D), obtaining no changes in GluN2B expression under glutamate or SNP stimuli by WB analysis (Figure 3B and 3C respectively). Finally, we analysed GluN2B expression by PCR in the cells treated with glutamate or SNP versus the control ones. As expected, there were no changes in GluN2B mRNA in any experimental condition (Figure 3E).

### 5′UTR represses the GluN2B translation and NO is able to derepress its translation

The observation that increased p-eIF2α correlates with an enhancement of GluN2B translation (Figures 2A, 2B) indicates that GluN2B has a different regulation than global proteins, which stop their translation when eIF2α is phosphorylated.

**Figure 4:**
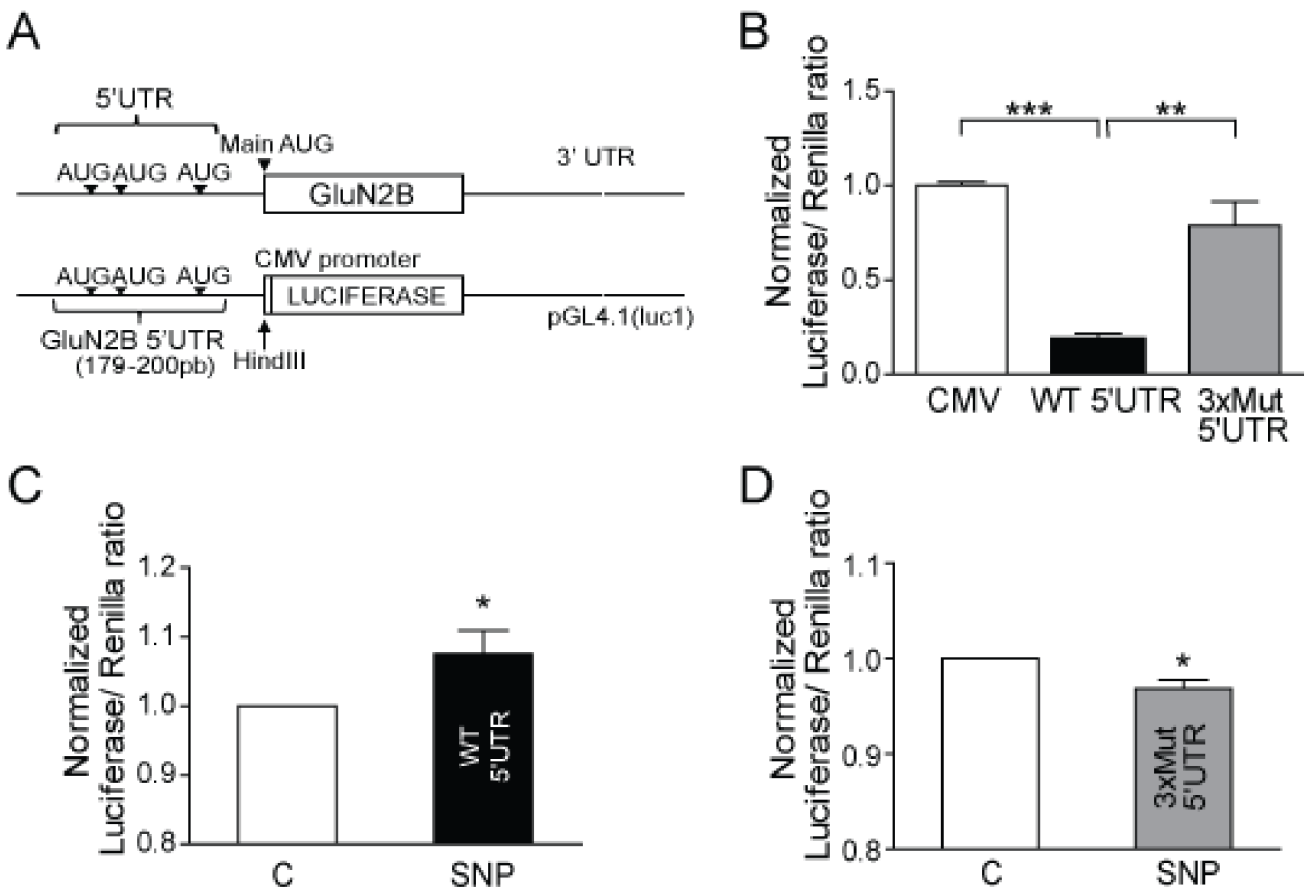
*GluN2B* 5′UTR repress GluN2B translation by its uAUG **A.** Representative scheme of *GluN2B* mRNA and the cloning of *GluN2B* 5′UTR in a pGL4.1 vector containing the luciferase reporter gene under CMV promoter control. **B.** Human neuroblastoma cells were transfected with 25 ng of the control pGL4.1 vector with CMV, with the WT 5′UTR of GluN2B or with the triple AUGs mutation (3xMUT). Data are the mean ± SEM of 3 independent experiments. **p < 0.001 ***p < 0.0001 one-way ANOVA with Bonferroni's post-test. **C.** Luciferase expression in human neuroblastoma cells transfected with the vector with the WT 5′UTR and untreated or treated with 100 nM SNP for 1 h. **D.** Luciferase expression in human neuroblastoma cells transfected with the vector with 3xMUT 5′UTR and untreated or treated with 100 nM SNP for 1 h. Data are the mean ± SEM of 4 independent experiments. *p < 0.05 by Student *t*-test.

There are a group of proteins whose mRNA have several uAUGs in the ORF in the 5′UTR of the mRNA sequence, as previously reported for some proteins [18, 23-25], and it makes the translational control of these proteins completely different. Interestingly the mRNA of GluN2B has a 5′UTR expanding 179 nucleotides with three uAUGs (access number in NCBI: NM_000834). Therefore we studied if the 5′UTR sequence was able to repress the GluN2B expression in rest conditions and if the presence of the 3 upstream AUGs were responsible (Figure 4). We cloned the 5′UTR of GluN2B (WT 5′UTR) in a vector upstream of a reporter luciferase gene under the control of a CMV promoter and expressed in neuroblastoma cells. We also generated a mutant of the three uAUG (3xMut), exchanging A for U, and a control vector that just express the luciferase gene and the CMV promoter (CMV) (Figure 4A).

Cells transfected with the WT 5′UTR-containing construct showed reduced luciferase expression compared to cells transfected with the vector without the 5′UTR (*p* < 0.0001; Figure 4B). However, the mutation of the three uAUGs reversed the effect of the 5′UTR in the repression of luciferase expression (*p* < 0.001; Figure 4B). Furthermore, we found a significant increase in luciferase expression in cells transfected with the WT 5′UTR vector after treatment with 100 nM SNP (*p* < 0.05; Figure 4C) but not in cells transfected with 3xMut (Figure 4D), which lack uAUGs completely. These findings suggest that the 3 uAUGs in the ORF of the *GluN2B* mRNA are the target for the p-eIF2α action.

### HRI kinase is activated by NO and phosphorylates eIF2α

**Figure 5:**
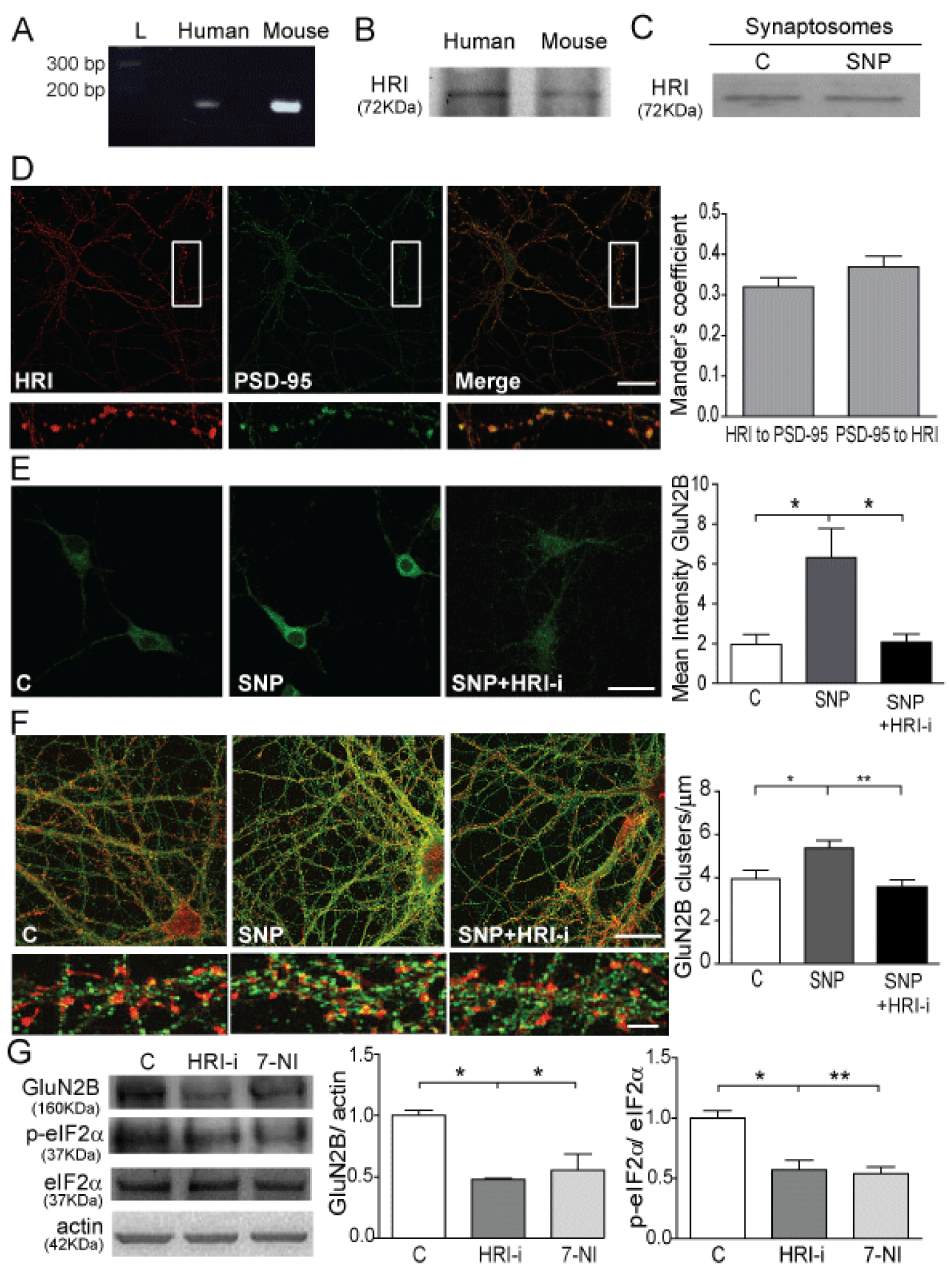
HRI kinase is involved in p-eIF2α and GluN2B translation **A.** PCR image showing HRI cDNA from mRNA in human cortex and mouse cortical neurons. **B.** WB of HRI immunoprecipitated from human cortex and mouse cortical neurons. **C.** Representative WB showing HRI presence in synaptosomes from mouse cortex untreated or treated with 100 nM SNP for 1 h. **D.** Confocal images showing co-localization (merge) between HRI (red) and PSD-95 (green). Quantification of the co-localization performed by Mander's coefficient analysis is shown (Scale bar: 10 μm). **E.** Confocal images showing GluN2B expression in control untreated neurons*,* treated with 100 nM SNP or treated with 100 nM SNP plus 1 μM HRI-i for 1 h. Quantification of the mean intensity of GluN2B is shown. Data are the mean ± SEM of 4 independent experiments. *p < 0.05 one-way ANOVA with Bonferroni's post-test (Scale bar: 20 μM). **F.** Confocal images from DIV21 cultures showing GluN2B (green) and phalloidin staining of actin (red) (Scale bar: 10 μM). Higher magnification of dendrites in the lower panels (Scale bar 5 μm). Quantification of GluN2B clusters were normalized by μm. Data are the mean ± SEM of 3 independent experiments. *p < 0.05 **p < 0.001 one-way ANOVA with Bonferroni's post-test. **G.** WB of synaptosomes from mice treated with DMSO, 50 mg/Kg HRI-i and 50 mg/Kg 7-NI. Quantification of data after correction for actin and total eIF2α is shown. Data are the mean ± SEM of 3 independent experiments. *p < 0.05, **p < 0.001 one-way ANOVA with Bonferroni's post-test.

There are four kinases involved in eIF2α phosphorylation: HRI, the Protein Kinase RNA activated (PKR), the double-stranded RNA-activated Protein-like ER Kinase (PERK), and the General Control Non-repressed 2 kinase (GCN2) [13, 16, 25, 26]. Among them, HRI has a heme group at the N-terminal domain that can bind NO. This action disrupts the intramolecular inhibitory interactions between the heme binding domain and the catalytic domain [27], activating HRI which is then capable of phosphorylating eIF2α. Therefore we hypothesized that HRI is responsible to producing the GluN2B translation in the glutamate-NO signalling cascade. Although HRI was first isolated from rabbit reticulocytes [28], it is also present in other cell types. We detected *HRI* mRNA (Figure 5A) and HRI protein (Figure 5B) in human cortex and cultured mouse primary cortical neurons, and also in mouse cortical synaptosomes, without changing its expression after NO stimulus (Figure 5C). We observed that HRI and PSD95 co-localizes in cortical neurons (Figure 5D). Mander's coefficient show that HRI co-localizes with PSD-95 at 30% and PSD-95 with HRI at 35% (Figure 5D).

To study the role of HRI in GluN2B expression we used a HRI inhibitor (HRI-i; [29]). First, we test the efficiency of the HRI-i in our model of study by a luminometric assay [30] (Supplementary Figure 2). To do this, we immunoprecipitate HRI kinase from mouse cortex and coincubate it with eIF2α substrate, ATP and luciferase substrate in a kinase buffer. The luminescence signal correlates with the amount of ATP present and it is inversely proportional to the amount of kinase activity. The graph shows we are able to immunoprecipitate the HRI kinase in an active stage (reduces a 60% of luciferase signal so the ATP presence) and the inhibitor 1 μM HRI-i in presence of the kinase is able to reduce the luciferase signal to only 40% compared to 60% when the HRI kinase is alone. The HRI-i at 1μM is able to reduce the HRI kinase activity by 33,33% (Supplementary Figure 2). Then, we obtained that the treatment with SNP plus HRI-i significantly prevents the GluN2B expression in cortical neurons (*p* < 0.05; Figure 5E). In order to verify that this control is working constitutively in neuronal function and if it is depending on developmental processes we studied the effect of NO in mature neurons (DIV21; Figure 5F). We obtained that NO is able to significantly increase the number of GluN2B clusters (*p* < 0.05; Figure 5F), but the coincubation with the HRI-i impairs this effect significantly (*p* < 0.001; Figure 5F). These findings demonstrate that HRI is constitutively working in neurons independent of the developmental stage

Finally, we intraperitoneally injected mice with HRI-i and 7-NI, a nNOS inhibitor, to observe the effect *in vivo* of these inhibitors in GluN2B expression and p-eIF2α levels (Figure 5G). As expected, cortical synaptosomes obtained from mice treated with HRI-i showed significantly lower levels of GluN2B and p-eIF2α compared to control animals treated with vehicle (DMSO) (*p* < 0.05; Figure 5G). The expression of GluN2B and the phosphorylation of eIF2α were also significantly reduced in mice treated with the nNOS inhibitor 7-NI (*p* < 0.05 and *p* < 0.001; Figure 5G), presumably due to the lower neuronal NO production and the inhibition of its downstream effects.

### Glutamate- and NO-induced GluN2B expression generate functional channels

**Figure 6:**
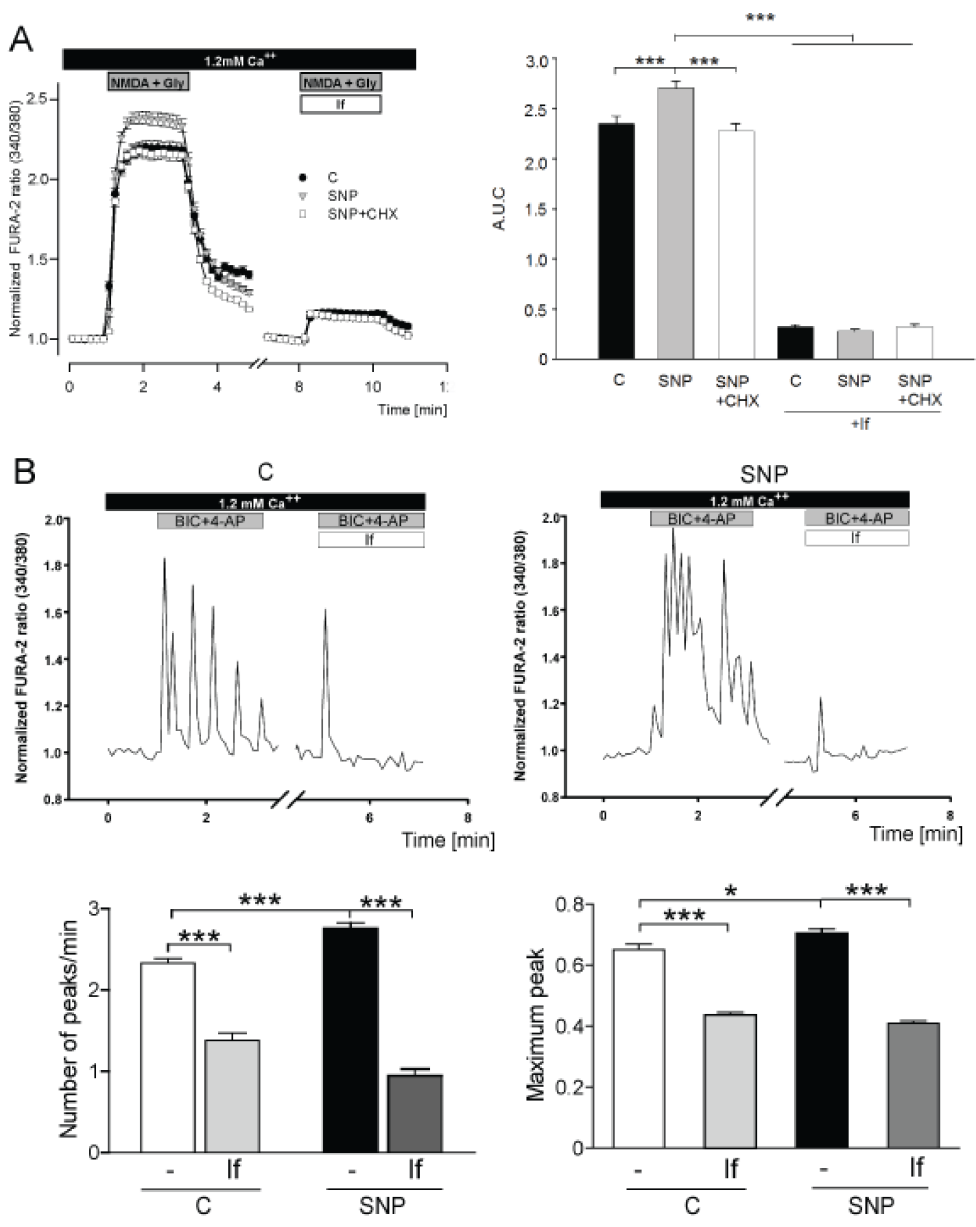
Effect of NO on GluN2B-mediated extrasynaptic and synaptic activity **A.** Measurement of Ca^2+^ entry into cortical neurons after stimulation with 100 μM NMDA plus 100 μM Gly for 2 min, in the absence/presence of 10 μM If. Control neurons (black circles), treated with 100 nM SNP (grey triangles) or 100 μM CHX plus 100 nM SNP (white squares) for 1 h. Quantification of the area under curve (A.U.C.) of each plot is shown. Data are the mean ± SEM of 6 independent experiments. ***p < 0.0001 by one-way ANOVA with Bonferroni's post-test. **B.** Representative plots showing the intracellular Ca^2+^ entry into cortical neurons treated for 1 h with 100 nM SNP, in response to stimulation with 50 μM BIC plus 2.5 mM 4-AP in the absence/presence of 10 μM If. Quantification of number of peaks and maximum peak in the experiments performed is shown. Data are the mean ± SEM of 6 independent experiments *p < 0.05 ***p < 0.0001 by one-way ANOVA with Bonferroni's post-test.

In order to know if the observed glutamate- and NO-induced increase in GluN2B expression produces functional channels, we performed calcium live-imaging experiments in cortical neurons (Figure 6). Stimulation with 100 μM NMDA plus 100 μM glycine (Gly) generated a significant Ca^2+^ entry in neurons pretreated 1 h with SNP compared to control untreated neurons (*p* < 0.0001; Figure 6A). The potentiating effect of SNP was abolished by CHX (*p* < 0.0001; Figure 6A). Quantitative analysis of the Ca^2+^ signal was accomplished by calculating the area under the curve (A.U.C.) as an indicator of the magnitude of the Ca^2+^ entry (Figure 6A, right panel). The observed Ca^2+^ response is specific for GluN2B-containing channels, as indicated by the blockage of NMDA-generated Ca^2+^ signal with the selective antagonist Ifenprodil (If) (Figure 6A).

Since the NMDA plus Gly treatment can activate both synaptic and extrasynaptic GluN2B containing receptors, we repeated the experiments with bicuculine (BIC) (Figure 6B), an antagonist of GABA A receptors, plus 4-aminopyridine (4-AP), a selective potassium channel blocker, in order to study the spontaneous synaptic activity [31] by blocking the inhibitory response and enhancing the membrane depolarization. Pretreatment with SNP shows that BIC plus 4-AP stimulation significantly increased the number and maximum peak of Ca^2+^ spikes (*p* < 0.0001 and *p* < 0.05; Figure 6B). This increase was mainly due to the action of the GluN2B subunit, as evidenced by the fact that the treatment with If significantly reduced both the number of peaks/minute and the maximum peak amplitude in control neurons and those treated with SNP (*p* < 0.0001; Figure 6B). Besides to the functional calcium experiments, we measured the co-localization of GluN2B subunits with GluN1, an essential subunit in active NMDA receptors (Supplementary Figure 1B). We observed that GluN2B-GluN1 co-localization is increased in dendrites of SNP treated neurons compared with the controls as quantified by the Mander's coefficient. These data support the inclusion of GluN2B in functional NMDA receptors.

### HRI is important for memory retrieval in mice

**Figure 7:**
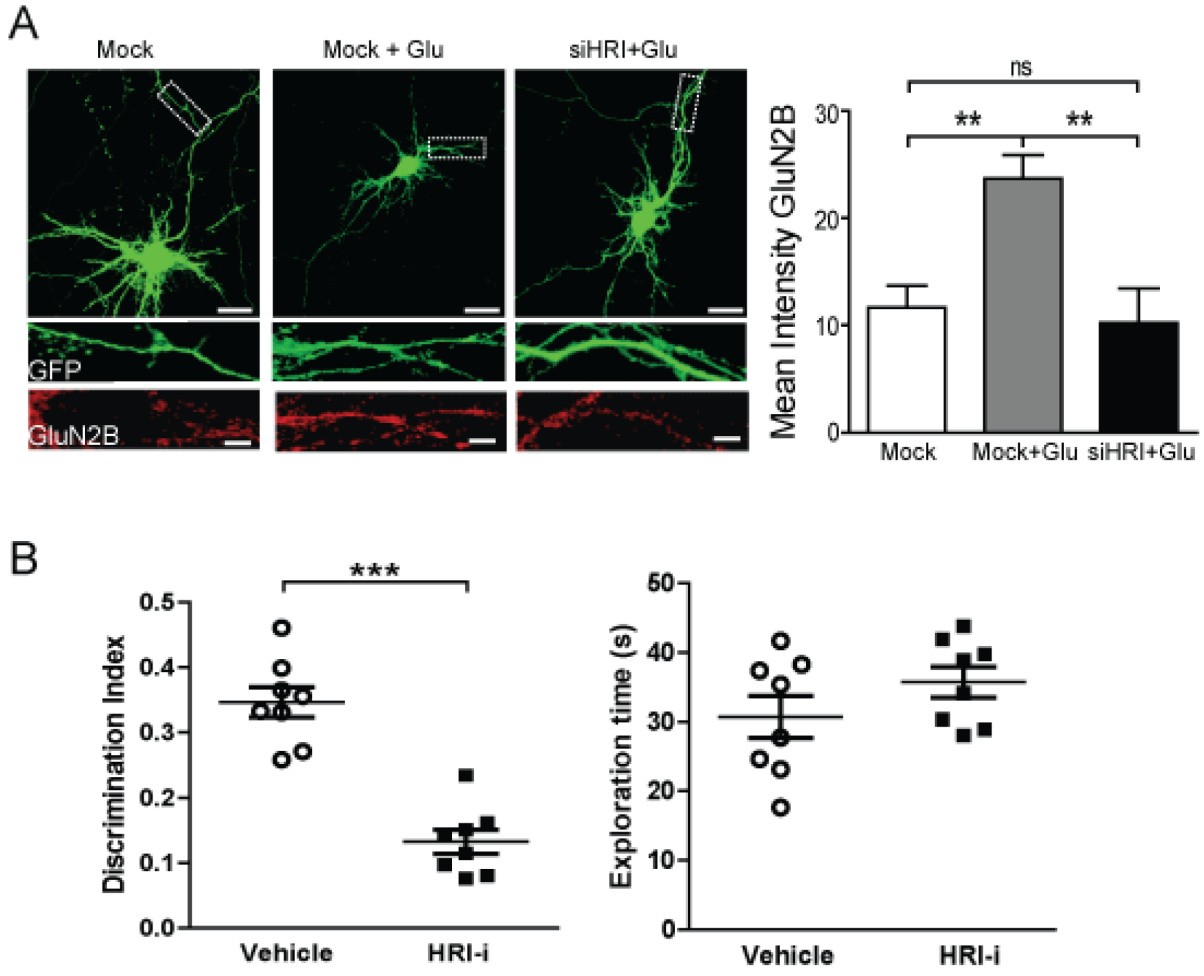
Functional effects of HRI ablation in cells and HRI inhibition in memory retrieval **A.** Immunofluorescence study of GluN2B expression in primary cortical cultures transfected with eGFP, under basal conditions (left panel) or after glutamate (10 μM) stimulation (center and right panels). Representative images of cortical neurons transfected with eGFP (green channel) with HRI siRNA (siHRI, right panel) or without (Mock). The insets represent the dendritic processes showing the endogenous levels of GluN2B subunit (red channel) after the treatments. Scale bars: 20 μm (low magnification) and 5 μm (inset). Statistical analysis using a one-way ANOVA with Bonferroni's post-test (**p < 0.01). **B.** Memory retrieval was impaired in mice treated with an HRI-i compared to vehicle. Discrimination index values were obtained in mice treated with the HRI-i 1 hour before the test session. Data are the mean ± SEM *n* = 8 mice per experimental group. ***p < 0.001 by Student *t-*test. No differences were observed in the total exploration times between the experimental groups.

Our data show that HRI modulates GluN2B expression and HRI inhibition will have dramatic consequences on neuronal function. Therefore we have performed a transient knockdown of endogenous HRI to reinforce the data obtained with the HRI-i. Cortical neurons were co-transfected with a HRI small interfering RNA (siHRI) and the eGFP plasmid for further detection of transfected neurons. Three days after transfection, cells were treated with 10 μM glutamate (or vehicle) for 60 min. The immunofluorescence study (Figure 7A) showed that GluN2B levels increased in glutamate treated cells but this increase was prevented in the cells co-transfected with the HRI siRNA, confirming the same results that we have obtained in the experiments using the HRI-i compound.

Given the important role of NMDAR in learning and memory, and to confirm the physiological relevance of the results obtained in cells and synaptosomes, we used an object-recognition task to evaluate the effects of HRI-i on memory retrieval, a key ability in global memory function. Mice receiving HRI-i shown memory impairment in the object recognition (D.I. = 0.13, *p* < 0.001) (Figure 7B). Such strong consequence of HRI inhibition was not related to locomotor effects, since the exploratory behavior, measured as the total exploration time in the memory test was not affected (Veh: 30.73 ± 3.03 sec; HRI inh: 35.73 ± 2.19 sec.; Figure 7B).

## DISCUSSION

NMDAR is an important player in synapses and therefore in learning and memory. Its expression must be tightly controlled since excess activity can trigger neuronal excitotoxicity and neurodegeneration [32, 33], while insufficient activity can produce neuronal pathologies such as cognitive and memory deficit [34, 35]. Furthermore, an abnormal low expression of NMDAR has been associated with schizophrenia [36, 37]. In particular, the GluN2B subunit of NMDAR is involved in neuronal growth and plays a role in synaptic plasticity [38]. GluN2B expression is highly regulated at the translational level [39], as the associated mRNA has a long 5′UTR that contains three uAUGs.

Protein synthesis is key to the consolidation of memory and learning. Transcriptional activation of genes regulated by the cAMP response binding-element transcription factor is required to promote the growth of the dendrites and the synaptic spines [1]. However, transcription is a time-consuming process, and for neuronal plasticity a fast rate of protein synthesis is required. For this reason, several mRNAs are transported into dendrites, where polyribosomes are ready to translate new proteins needed immediately after synaptic stimulation [40-42]. It has been reported that mRNAs of GluN2A, and especially GluN2B, are located in neurites [43, 44]. Therefore, we propose that glutamatergic signalling can induce the rapid expression of GluN2B subunits in dendrites shortly after translational activation. Once expressed, the GluN2B subunit can assemble with the GluN1 subunit, which is synthesized in excess (estimated ≈ 10-fold compared to GluN2 [45, 46], to form active receptors. The GluN2B subunit is critical to allow a functional NMDAR and the tight control of its expression is based in *GluN2B* mRNA 5′UTR containing 3 uAUGs that are able to repress the translation of GluN2B in conditions that do not require a special synaptic activation.

**Figure 8:**
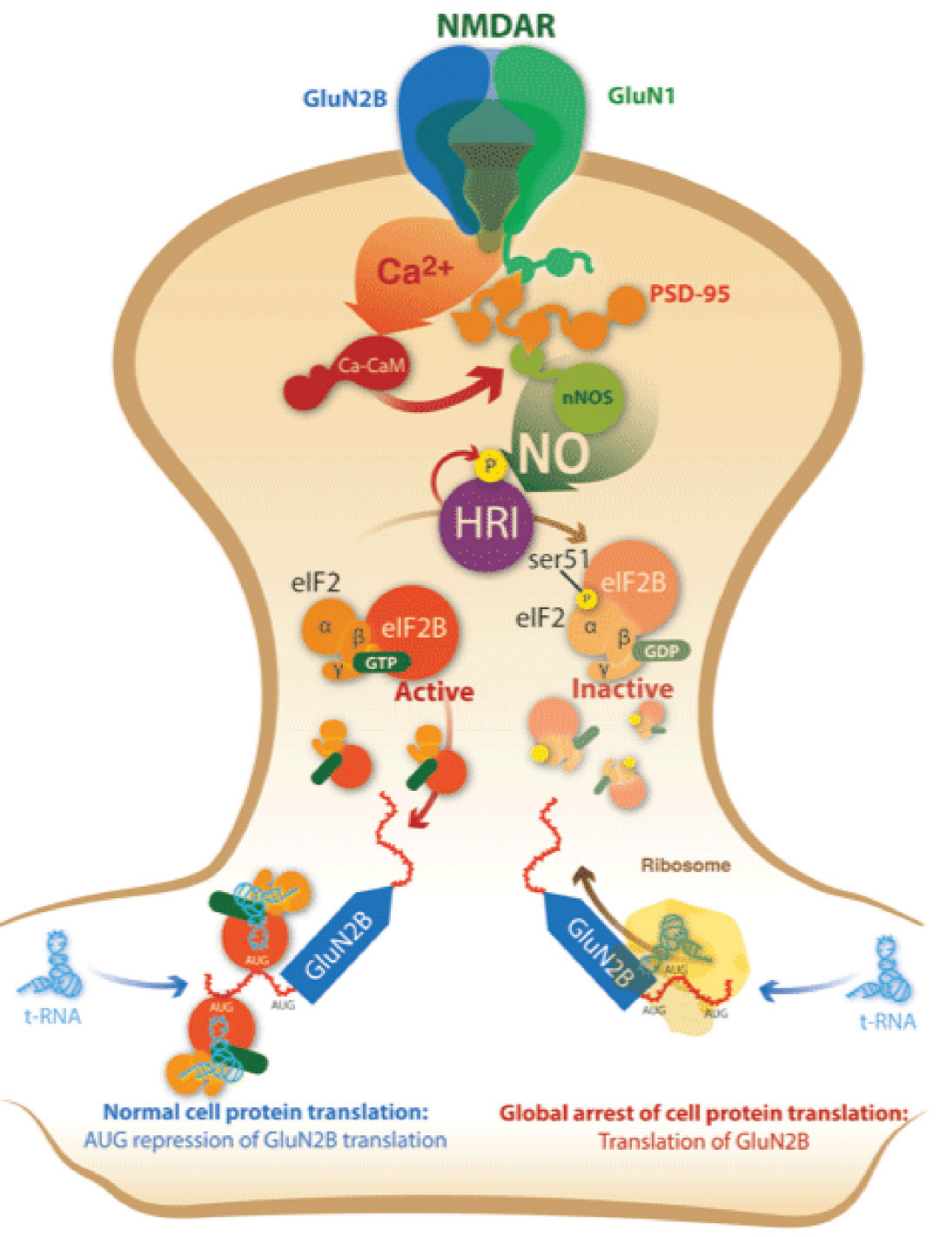
Signalling pathway for glutamate-NO-HRI-eIF2α inducing GluN2B translation at postsynaptic ending Glutamate binds NMDAR that allows a flow of Ca^2+^ making nNOS to produce NO. NO activates HRI that phosphorylates eIF2α, and its phosphorylation induces a global arrest of the cell protein translation but allows the translation of these mRNAs bearing several AUGs in its 5′UTR such as GluN2B mRNA.

To allow the rapid expression of GluN2B upon glutamate-NO activation, HRI phosphorylates eIF2α, arresting the global protein translation but activating those bearing several uAUGs in their ORF (Figure 8). HRI assumes the main function of providing new functional NMDAR, allowing synaptic plasticity. This mechanism further supports the link of HRI with learning processes in brain [19] and with the spine growth formation [18]. In fact, we obtained that the injection of HRI-i in mice produced a decrease in the memory retrieval.

PKR, PERK, and GCN2 are also capable of eIF2α phosphorylation. However, as these kinases are activated under stress conditions, they would likely contribute to an increased GluN2B expression that would trigger glutamatergic excitotoxicity. Regarding this point, recent evidence [47] demonstrates an increase of the eIF2α phosphorylation in Alzheimer's disease patients.

Our functional assays measuring changes in intracellular [Ca^2+^] triggered by direct NMDA stimulation or increased synaptic activity with BIC plus 4-AP showed that neurons pretreated with a NO donor allowed greater Ca^2+^ entry, indicating that the increase in GluN2B translation results in an increase in NMDAR-mediated response. The relevance of these findings is that the proposed signalling pathway is providing a very rapid mechanism to amplify the glutamate signalling by the generation of new functional receptors upon the required physiological stimulation.

In summary, we propose a novel regulatory mechanism for GluN2B subunit expression in glutamatergic signalling, which might have important physiological implications in memory and learning processes since these functions are highly dependent on glutamate.

## MATERIALS AND METHODS

### Ethics statement

Handling and investigation with the human samples has been carried out in compliance with the ethical standards and according to the Helsinki Declaration and to the Ethics Committee of the Institut Municipal d’Investigacions Mèdiques-Universitat Pompeu Fabra (EC-IMIM-UPF).

### Embryo neuronal cell cultures

This was carried out according to the procedure reported previously [16]. Neurons were isolated from 18 day-old mice. Cortex tissue was aseptically dissected and trypsinized. Cells were seeded on 1% poly-L-Lysine (Sigma-Aldrich, USA) coated coverslips (7.5 × 10^4^cells/cover) and cultured in DMEM high glucose (Gibco, USA) and 10% horse serum (Gibco). After 2 h, the medium was removed and DMEM high glucose medium containing 1% B27 supplement (Gibco) plus antibiotics was added. In the third day of culture, 2 μm of Arabinoside C was added to avoid the glial cells grow. Cultured neurons were used for Ca^2+^ experiments and for western blot (WB) and immunofluorescence microscopy on *day* *in vitro* (DIV) 10.

### Human brain samples

Human brain tissue sample was supplied by the Banc de Teixits Neurològics (Neurological Tissue Bank of the Biobank-Hospital Clínic-IDIBAPS, Barcelona, Spain), the Unitat d’Anatomia Patològica (Hospital del Mar) and the Unitat de Neuropatología y Banco de Cerebros (Fundación Hospital Alcorcón). The human brain sample was obtained from the frontal cortex of a healthy aged individual. The sample was lysed with a cocktail containing NP40 lysis buffer (150 mM NaCl, 5 mM EDTA, 1% Nonidet P-40, 1mM sodium orthovanadate, 1mM phenylmethylsulphonyl fluoride, 0.05% aprotinin, 1mM dithioltreitol) and a 1x of protease inhibitors (Complete mini-EDTA free, Roche Diagnostics GmbH, Switzerland). The mixture was mechanically disaggregated and centrifuged at 17,500 x g for 10 min. The supernatant was quantified by the Bicinchoninic Acid (BCA; Thermo Scientific, USA) assay.

### Mice treatments for synaptosome preparations

The HRI inhibitor (HRI-i; *N*-(2,6-dimethylbenzyl)-6,7-dimethoxy-2H-[1]benzofuro [3,2-c]pyrazol-3-aminehydrochloride; Janssen Research & Development; La Jolla, USA; 50 mg/kg), the nNOS inhibitor (7-NI; Sigma-Aldrich; 50 mg/Kg) and vehicle control (DMSO; Sigma-Aldrich) were administered intraperitoneally (i.p.) in a volume of 2 mL/Kg in C57BL/6 (Charles River, France) mice. The procedure was approved by the EC-IMIM-UPF.

### Preparation of cortical synaptosomes

Cortical synaptosomes from the cortex of C57BL/6 mice were obtained as previously described [48]. Synaptosome integrity was assessed by electron microscopy (Figure 1C).

### Subcellular fractionation of primary cortical cultures

Subcellular fractionation was conducted on primary cortical cultures at DIV10, previous treatment with the NO donor 100 nM sodium nitroprusside (SNP; Sigma-Aldrich), using an adapted protocol [49]. The samples were resolved by WB using the following antibodies: anti-GluN2B (Sigma, Cat. N. M-265); anti-PSD-95 (Neuromab Cat. N. 75-028); anti-synapsophysin (SYP)(Sigma, Cat. N. S5768).

### Cloning of GluN2B 5′-untranslated region (UTR)

Total RNA was extracted from hippocampus tissue and one-step RT-PCR was carried out using primers designed to amplify GluN2B 5′UTR: 5′-CATTTATCCTTCGTCTTTCTTATGTG-3′,5′-CAACACCAACCAGAACTTG- 3′. The PCR product, a single band matching the molecular weight of GluN2B 5′UTR (∼180 nt), was isolated and purified from agarose gel using the Ilustra™ GFX™ PCR DNA and Gel Band Purification kit (GE Helthcare, UK) and stored at -20°C. The 5′UTR DNA fragment was then inserted into the HindIII site of a modified pGL4.10 [luc2] vector (Promega, USA) containing the CMV promoter cloned at BglII and HindIII sites.

### Generation of GluN2B 5′UTR mutants

5′UTR mutations were generated by site-directed mutagenesis (QuikChange^®^ II XL site-directed mutagenesis kit, Stratagene). The A from the first and second ATG were substituted with T with the primers: 5′-CCTTCGTCTTTC TTTT GTGGATTTGCAAGCGAGAAGAAGGG-3′ and 5′-CCCTTCTTCTCGC TTGCAAATCCACAAAAGAAAGACGAAGG-3′. This construct was used as the template for exchange of A from the third ATG with T in the following primers: 5′-CTGGACATT CCCAACTTGCTCACTCCCTTAATCTG-3′ and 5′-CAGATTA AGGGAGTGAGCAAGTTGGGAATGTCCAG-3′. The incorporation of the mutations was verified by sequentation.

### Transient DNA transfection of SH-SY5Y cells and luciferase assay

SH-SY5Y neuroblastoma cells were seeded in 96-well plates at a density of 15,000 cells/well and grown for 12 h with DMEM plus 10% fetal bovine serum (Gibco). Afterwards, a total of 250 ng DNA was transfected into each well according to the following conditions: 250 ng pcDNA3 plasmid as blank, 25 ng renilla plus 25 ng CMV-luciferase vector plus 200 ng pcDNA3, and finally, 25 ng renilla plus 25 ng GluN2B-5′UTR CMV-luciferase construct plus 200 ng pcDNA3 as experimental samples. Cells were transfected using JetPEI transfection reagent (PolyPlus, Korea) for 4h. The medium was then exchanged and cells were incubated for 24 h to allow sufficient gene expression. After 1 h of treatments with the NO donor SNP cells were lysed and luciferase and renilla activities were measured by using the Dual-Glo™ Luciferase Assay System (Promega) following manufacturer's instructions. The luminescence was read using the plate luminescence reader (Fluostar OPTIMA, BMG labtech, Germany).

### Cellular and synaptosome treatment

100 nM SNP and 10 μM L-glutamate (Sigma-Aldrich) were added to cortical neuronal cultures and synaptosomes, and maintained for 1 h at 37°C. 10 μM 7-NI, 100 μM cycloheximide (CHX), 1 μM Actinomycin D (Act D) and 1 μM HRI-i, and 10 μM BAPTA·AM (a calcium quelator), were pre-incubated for 30 min and then the inhibitors were incubated with the stimulators SNP, and glutamate for 1 h at 37°C.

### Extraction of mRNA and RT-PCR

RNA extraction (Nucleospin RNA II kit, Macherey-Nagel) from human cortex and mouse cortical neuron was carried out and RT-PCR was performed using SuperScrip-RT system (Invitrogen). Aliquots of 1 μg cDNA were used as a template for PCR. The primers used to amplify HRI from humans were: 5′-CCCCGAATATGACGAATCTG-3′ and 5′-CAGATTCGTCATATTCGGGC-3′; the primers used to amplify HRI from mouse were: 5′-GAAGTGGGTTTGGTTCATGC-3′ and 5′-GCATGAACCAAACCCACTTC-3′; the primers to amplify GluN2B from mouse were: 5′- CAACACCAACCAGAACTTG-3′ and 5′- CATTTATCCTTCGTCTTTCTTATGTG-3′; the primers to amplify GAPDH from mouse were: 5′-TGTCGTGGAGTCTACTGGTGTCTT and 5′- TGGCTCCACCCTTCAAGTG. PCR conditions for all transcripts were: 95°C for 3 min; 95°C for 30 s; 60° for 30 s, 72°C for 30 s; 72°C for 7 min; with 35 cycles of amplification. The amplified HRI, GluN2B, and GAPDH were resolved in a 2% agarose gel.

### Protein levels detection by WB

Cortical cells were washed twice with PBS, detached mechanically with a scraper, and lysed with NP40 lysis buffer and a 1x of protease inhibitors (Complete mini-EDTA free from Roche Diagnostics GmbH). Samples (100 μg) were resolved in 8% polyacrilamide gels. Gels were transferred in polyvinylidene fluoride membranes (ImmobilonP, Millipore, USA), and blocked for 1 h to with a tween-tris buffer saline (TTBS)-5% milk. Membranes were incubated overnight (ON) at 4°C with the primary antibodies (Abs). Primary Abs were incubated with the following mixtures: mouse GluN2B Ab (Neuromab, USA) 1:5 in TTBS; rabbit phospho-eIF2α (p-eIF2α Ab) (Abcam, UK) 1:500 in TTBS-5% bovine serum albumin (BSA); mouse eIF2α total Ab (Abcam) 1:500 in TTBS-5% milk; rabbit HRI Ab (Abcam) 1:1000 in TTBS-5% milk; mouse actin Ab (Sigma-Aldrich) 1:4000 in TTBS-5% milk; Membranes were washed thrice with TTBS to release the excess of antibody. Anti-mouse and anti-rabbit secondary Abs (GE-Healthcare) at 1:4000 dilutions with TTBS-5% milk were respectively added in membranes and stirred for 1 h. Three washes with TTBS were performed. Membranes were revealed with Supersignal West Pico and Femto Chemiluminiscent substrate (Thermo Scientific Pierce). Blotting quantification was done with Quantity One software. GluN2B and p-eIF2α band intensity were normalized by actin and eIF2α total levels, respectively.

### Immunofluorescence experiments and colocalization analysis

Mouse cortical cells were seeded at 75,000 cells/well on poly-L-lysine (Sigma-Aldrich) coated coverslips. Cells were fixed with 4% paraformaldehyde (PFA) for 10 min and washed three times with PBS (10 min each) to remove PFA traces. Cells were permeabilized with 0.1% Triton X-100 and washed thrice with PBS. Coverslips were incubated with blocking solution (5% fetal bovine serum, 1% BSA and 0.02% sodium azide) over night at 4 °C. Subsequently, cells were incubated for 2 h at room temperature (RT) in a hydration chamber with 1:10 mouse anti-GluN2B Ab (Neuromab), 1/700 rabbit anti-GluN2B Ab (Abcam), 1/500 mouse anti-PSD95 Ab (Cell signalling), 1/500 rabbit anti-HRI Ab (Abcam) or 1/500 mouse anti-GluN1 Ab (Synaptic system). After primary Ab incubation, cells were incubated with 1:1000 Alexa Fluor 488 goat anti-mouse Ab or 1:1000 Alexa Fluor 565 goat anti-rabbit Ab (Invitrogen) for 1 h at RT and washed three times (5 min each) with PBS. Digital images were taken with a Leica TCS SP confocal microscope and analysed with Leica confocal software (Heidelberg, Germany). Co-localization analyses were performed with Image J software, adjusting the threshold of 10 dendrites from 6 different neurons for each experimental condition from 3 independent experiments.

### HRI siRNA transfection in primary cortical neurons

Transient knockdown of endogenous HRI was achieved by co-transfecting primary cortical neurons at DIV11 (using Lipofectamine^™^ 2000, Life Technologies) with siRNA targeting HRI (GeneSolution siRNA, Qiagen) and the eGFP plasmid for further detection of transfected neurons. Three days after transfection, cells were treated with 10 μM glutamate (or vehicle) for 60 min and then fixed in a PBS solution containing 4% PFA and sucrose 4%. Further immunodetection of total GluN2B subunit was achieved as previously described.

### GluN2B clustering detection

Cortical neurons in DIV21 were treated with 100 nM SNP in the absence and presence of HRI-i for 1 h. Cells were fixed and incubated with anti-GluN2B Ab as previously described, and actin was detected by phalloidin-red (1/2500). Images were obtained with a Zeiss Axiovert 200M LSM PASCAL Confocal Laser Scanning Microscope. High-resolution (2,048×2,048 pixel) fluorescence images were acquired with a LP530 emission filter and excitation at 488 nm. The analysis of the cluster was performed with the pluggin analyze particles from ImageJ software.

### HRI immunoprecipitation from human brains and cortical neurons

Homogenated brain and cortical neurons protein (200 μg) were used for the immmunoprecipitation (IP). The samples were pre-incubated 30 min at 4 °C with protein G (GE Healthcare), which had been previously washed with PBS (this step is needed to avoid unspecific protein binding with protein G). The samples were then centrifuged at 10,000 x g for 10 min. ON incubation of SN with 5 μg anti-HRI Ab (Abcam) was followed by the addition of protein G immobilized on sepharose (GE Healthcare) and shaking for 2 h at RT. HRI was then pelletized by centrifugation at 14,000 x g for 10 min and washed thrice. 60 μL loading buffer (x5) were added to pellets followed by boiling for 6 min at 100°C. Immunoprecipitated HRI was recovered by centrifugation at 14,000 x g for 10 min and 30 μl of the immunoprecipitated HRI were resolved by WB.

### Measurement of intracellular [Ca^2+^] in cortical mouse neurons

Cytosolic Ca^2+^ signal was determined at RT in cells loaded with 4.5 μM FURA-2·AM (Molecular probes Life technologies) (30 min) as previously described [50]. Cytosolic Ca^2+^ increases are represented as the normalized ratio of emitted fluorescence (510 nm) after excitation at 340 and 380 nm, relative to the ratio measured prior to cell stimulation (FURA-2 ratio 340/380). Cells were bathed in an isotonic solution (ISO) containing (in mM): 140 NaCl, 5 KCl, 1.2 CaCl_2_, 0.5 MgCl_2_, 5 glucose, 10 HEPES (300 mosmol/L, pH 7.4 with Tris). The following reactives were used during calcium experiments: 10 μM threo ifenprodil hemitartrate (If; a selective inhibitor of NMDA receptor having a GluN2B subunit; Tocris), 100 μM NMDA plus 100 μM Gly, 50 μM-(-) bicuculine methiodide (BIC; an antagonist of GABA A receptor; Tocris) plus 2.5 mM 4-aminopyridine (4-AP; a selective blocker of potassium channel Sigma).

### HRI kinase activity assay

The inhibition of HRI by the HRI-i was determined using a luminometric HRI activity assay. This method has been previously described to evaluate the activity of other inhibitors against other kinases like GSK3-β [30]. Briefly, HRI-i was tested at 1 μM (the concentration used in all the experiments) diluted in kinase buffer (containing final concentrations of: 20 mM Tris pH 8, 50 mM KCl, 25 mM MgCl_2_ and 1μM phenylmethysulfonil fluoride) and in presence of 5 μM. eIF2α peptide substrate (Santa Cruz Biotechnology), immunoprecipitated HRI from mouse cortex (goat, Santa Cruz Biotechnology) and 10 μM ATP, to a total assay volumen of 50 μl. The enzymatic reaction was stopped after 5 min of incubation at room temperatura by adding 50μL of Kinase Glo (Promega). After 10 min of stabilization, the luminiscence was measured with a Turner Designs Luminometer Model TD20/20.

### Novel object recognition test

Adult male C57BL/6J mice were used. Mice were trained and tested in the V-maze for a novel object-recognition memory, as previously described [51]. Object-recognition memory was tested 24 h after the training session and 1 h after HRI-i (15 mg/kg) or its vehicle (2% DMSO, 3% Ethanol, 5% Cremophor, 90% saline) were injected intraperitoneally (i.p.) in a volume of 10 ml/kg, in order to study memory retrieval. A discrimination index (DI) was calculated based on the time of exploration for the novel (Tn) and the familiar (Tf) objects in the test session (DI = (Tn-Tf)/(Tn+Tf)), as previously described [51]. The total object-recognition exploration time (Tn+Tf) was used as a measure of activity to discard acute locomotor effects of the treatment.

### Statistical analysis

Data were expressed as the mean ± SEM of the values from the number of independent experiments as indicated in the corresponding figure legend. Data were evaluated statistically by using one-way ANOVA with Bonferroni's and Newman-Keuls Multiple Comparison post-test for multiple comparisons and Student's *t*-test for comparisons between two conditions (*p* < 0.05 was considered significant). The software used was Graph Pad Prism version 5.00 for Windows, GraphPad software, San Diego California USA,  www.graphpad.com and Sigma Plot for Windows, version 12.00 build 12.2.0.45. copyright© 2011 Systat Software Inc.

## SUPPLEMENTARY MATERIAL FIGURES



## Abbreviations

NMDAR: N-Methyl D-Aspartate Receptor
NO: nitric oxide
eIF2α: eukaryotic translation initiation factor 2 α
HRI: heme-regulated eIF2α kinase
PSD-95: postsynaptic density-95
nNOS: neuronal NO synthase
5′UTR: 5′ untranslated region
uAUG: upstream AUG
ORF: open reading frame
Ser: serine
DIV: day *in vitro*
Ab: antibody
IP: immunoprecipitation
RT: room temperature
ISO: isotonic solution
PKR: Protein Kinase RNA activated
PERK: the double-stranded RNA-activated Protein-like ER Kinase
GCN2: General Control Non-repressed 2 kinase.
