# Supplementary material for: Glutamatergic stimulation induces GluN2B translation by the nitric oxide-Heme-Regulated eIF2α kinase in cortical neurons
Source: Oncotarget. 2016 Aug 19;7(37):58876–92. doi: 10.18632/oncotarget.11417 (PMC5312282; doi:10.18632/oncotarget.11417)
Supplement: Supplementary file 1 [file oncotarget-07-58876-s001.pdf]

Glutamatergic stimulation induces GluN2B translation by the nitric oxide-Heme-Regulated eIF2α kinase in cortical neurons

Supplementary Material

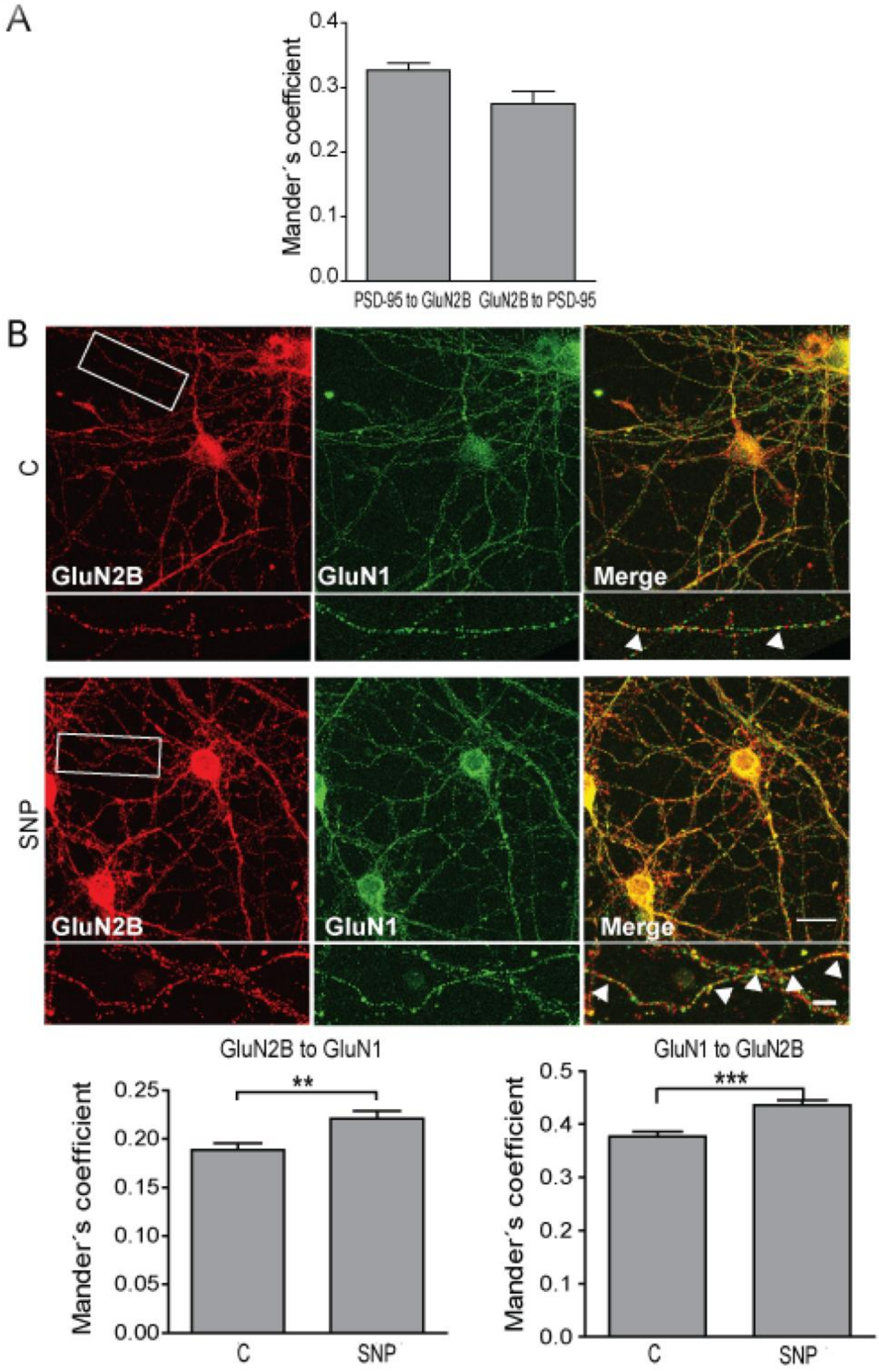

Supplementary Figure 1. A. Mander's coefficient between PSD-95 to GluN2B. Quantification of colocalization between PSD-95 and GluN2B from experiment of Figure 1A. **B.** Colocalization between GluN2b and GluN1 subunit of NMDA receptors in neurons untreated or treated with 100 nM SNP. The insets are representative images of dendrites showing endogenous levels of GluN1 or GluN2b. Scale bar:20 µm (low magnification), 5 µm (inset). Quantification of colocalization by Mander's coefficient analysis. 10 dendrites from 6 different neurons were analysed from each condition for each independent experiment. Data are the mean ± SEM of 3 independent experiments **p<0.01; ***p<0.0001 by Student *t*-test.

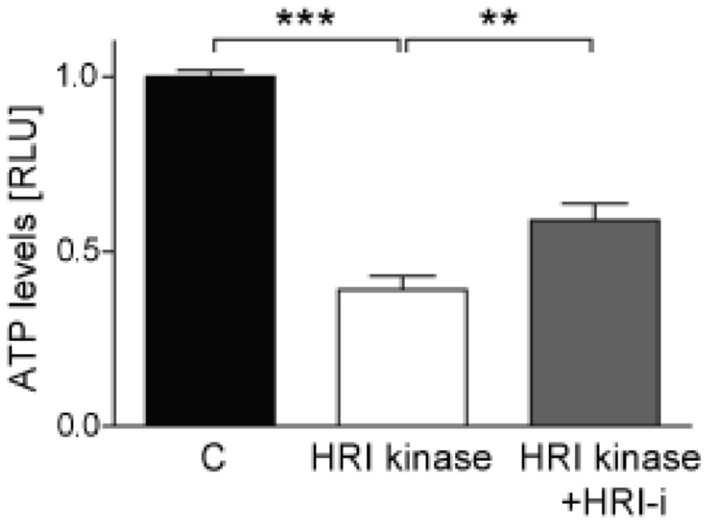

Supplementary Figure 2. HRI-i is able to inhibit HRI kinase activity. Representative graph of HRI activity and the inhibition of HRI by HRI-i by quantifying amount of remaining ATP in luminescence reaction. n=6-9 independent experiments. **p<0.001 ***p<0.0001 one-way ANOVA with Bonferroni´s post-test.
